# Hyperglycemia Induces Cellular Hypoxia through Production of Mitochondrial ROS Followed by Suppression of Aquaporin-1

**DOI:** 10.1371/journal.pone.0158619

**Published:** 2016-07-06

**Authors:** Kiminori Sada, Takeshi Nishikawa, Daisuke Kukidome, Tomoaki Yoshinaga, Nobuhiro Kajihara, Kazuhiro Sonoda, Takafumi Senokuchi, Hiroyuki Motoshima, Takeshi Matsumura, Eiichi Araki

**Affiliations:** 1 Department of Metabolic Medicine, Faculty of Life Sciences, Kumamoto University, Kumamoto, Japan; 2 Department of Molecular Diabetology, Faculty of Life Sciences, Kumamoto University, Kumamoto, Japan; Stellenbosch University, SOUTH AFRICA

## Abstract

We previously proposed that hyperglycemia-induced mitochondrial reactive oxygen species (mtROS) generation is a key event in the development of diabetic complications. Interestingly, some common aspects exist between hyperglycemia and hypoxia-induced phenomena. Thus, hyperglycemia may induce cellular hypoxia, and this phenomenon may also be involved in the pathogenesis of diabetic complications. In endothelial cells (ECs), cellular hypoxia increased after incubation with high glucose (HG). A similar phenomenon was observed in glomeruli of diabetic mice. HG-induced cellular hypoxia was suppressed by mitochondria blockades or manganese superoxide dismutase (MnSOD) overexpression, which is a specific SOD for mtROS. Overexpression of MnSOD also increased the expression of aquaporin-1 (AQP1), a water and oxygen channel. AQP1 overexpression in ECs suppressed hyperglycemia-induced cellular hypoxia, endothelin-1 and fibronectin overproduction, and apoptosis. Therefore, hyperglycemia-induced cellular hypoxia and mtROS generation may promote hyperglycemic damage in a coordinated manner.

## Introduction

Diabetes causes complications including retinopathy, nephropathy, neuropathy, and macroangiopathy. To minimize the risk of diabetic complications, blood glucose, blood pressure, and lipids should be properly controlled. However, although we now have various means of treating diabetes, there are still many patients who develop diabetic complications. Therefore, other approaches based on the elucidation of mechanisms of diabetic complications may be required to prevent diabetic complications.

We previously demonstrated that mitochondrial reactive oxygen species (mtROS) generation is the major cause of diabetes-induced oxidative stress, and that it causes other metabolic abnormalities, such as polyol pathway activation, advanced glycation end products formation, and protein kinase C activation [1]. The “hyperglycemia-induced mtROS generation” hypothesis may be one of the prevailing theories in the pathogenesis of diabetic complications [2]. However, many unanswered questions remain on the mechanisms of diabetic complications.

Glucose is metabolized by glycolysis and the tricarboxylic acid (TCA) cycle, which yields ATP, NADH, and FADH_2_. Electrons from NADH and FADH_2_ are then transferred to molecular oxygen, and the energy released from these oxidation/reduction reactions is used to drive the synthesis of ATP from ADP during oxidative phosphorylation, also known as the electron transport chain cycle. Therefore, in the metabolism of glucose, oxygen is consumed. As increased glycolysis, TCA cycle, and oxidative phosphorylation were the source of hyperglycemia-induced mtROS generation [1], hyperglycemia may increase oxygen consumption in mitochondria, resulting in cellular hypoxia. Indeed, there are some common aspects between hyperglycemia and hypoxia-induced phenomena, such as overproduction of endothelin-1 and fibronectin, and induction of apoptosis [3, 4].

Therefore, we hypothesized that hyperglycemia could cause cellular hypoxia in endothelial cells, which is regarded as a target tissue in the pathogenesis of diabetic complications [5], and that the hyperglycemia-induced cellular hypoxia and mtROS generation may promote hyperglycemic damage in a coordinated manner.

## Materials and Methods

### Cell culture conditions and materials

Bovine aortic endothelial cells (BAECs) were purchased from TOYOBO (Osaka, Japan) and used in passages 2–6. Cells were cultured in Dulbecco’s modified Eagle’s medium (DMEM, Wako, Osaka, Japan) with 10% fetal bovine serum (FBS) and antibiotic-antimycotic mixed stock solution (Nacalai Tesque, Kyoto, Japan). Cells were maintained in 5% CO_2_, 95% air at 37°C and incubated with 5.5 mM glucose DMEM. For hypoxic conditions, cells were placed in a multi-gas incubator MCO-5M (SANYO, Osaka, Japan) that was flushed with 1% O_2_, 5% CO_2_, balance N_2_ at 37°C.

Bis-2-(5-phenylacetamido-1,2,4-thiadiazol-2-yl)ethyl sulfide (BPTES), rotenone, and antimycin A were from Sigma-Aldrich Japan (Tokyo, Japan). Hydrogen peroxide was from Santoku Chemical Industries (Tokyo, Japan).

BAECs were incubated for 16 h in 0.4% FBS before the experiments. During the experiments, cell survival was monitored using the Cell Counting Kit-8 (Dojindo Molecular Technologies, Tokyo, Japan) and no changes in cell viability were observed (data not shown).

### Adenoviral vectors

Human manganese superoxide dismutase (MnSOD; a specific SOD for mtROS) adenoviral vectors were provided by Dr. M. Brownlee (Albert Einstein College of Medicine, Bronx, NY) [1], and human aquaporin-1 (AQP1) adenoviral vectors were purchased from Applied Biological Materials (Richmond, BC, Canada). Cells were infected with MnSOD or AQP1 adenoviruses 48 h before the experiments. MnSOD or AQP1 overexpression in BAECs was confirmed by western blot analysis, as previously described [6].

### Detection of intracellular hypoxia

Detection of intracellular hypoxia was performed as previously described with slight modification [7]. Briefly, cells were cultured under indicated experimental conditions, and 3 h before the end of the experiments, the medium was changed to fresh DMEM containing 10 μM pimonidazole hydrochloride (Hypoxyprobe, Inc., Burlington, MA, USA). Cells were fixed with 4% formalin neutral buffer solution (Nacalai Tesque) for 30 min at room temperature. Fluorescein isothiocyanate (FITC)-labeled mouse monoclonal hypoxyprobe-1 antibody (1:100; Hypoxyprobe, Inc.) and Alexa Fluor 488 conjugated goat-anti-mouse immunoglobulin antibody (Molecular Probes, Eugene, OR, USA) were used as primary and secondary antibody, respectively. Fluorescence was detected with a laser scanning confocal microscope (Olympus FV1200; Olympus, Tokyo, Japan). The fluorescence values from randomly selected cells were analyzed with FV10-ASW Viewer Software (Olympus).

To detect hypoxia, we also used the hypoxia probe LOX-1 (SCIVAX Co., Kanagawa, Japan). Experiments were carried out according to the manufacturer’s instructions. Briefly, cells were cultured under experimental conditions for 24 h with 2 μM LOX-1 and observed by a laser scanning confocal microscope. The fluorescence values from randomly selected cells were analyzed with FV10-ASW Viewer Software.

### Measurement of mitochondrial ROS generation and oxidative stress *in vitro*

To evaluate the direct generation of mitochondrial ROS, we used the reduced MitoTracker Red probe (CM-H_2_XRos) (M-7513; Molecular Probes) [8]. Briefly, cells were cultured under experimental conditions, and incubated with 300 nM CM-H_2_XRos at 37°C for 15 min before the end of the experiment. Cells were fixed with 3.7% paraformaldehyde (Nacalai Tesque) in Hanks' balanced salt solution (HBSS, Nissui Pharmaceutical Co., Ltd., Tokyo, Japan) for 15 min at room temperature. In addition, we investigated the effect of AQP1 overexpression on oxidative stress *in vitro*. Cells transduced with AQP1 or control adenoviruses were incubated for 24 or 96 h under indicated conditions, and then cells were fixed with 4% formalin neutral buffer solution (Nacalai Tesque) for 30 min at room temperature. 8-OHdG (8-hydroxy-2'-deoxyguanosine) mouse monoclonal antibody 15A3 clone (1:100; Santa Cruz Biotechnology Inc., Santa Cruz, CA, USA) and Alexa Fluor 488 conjugated goat-anti-mouse immunoglobulin antibody (Molecular Probes) were used as primary and secondary antibody, respectively. A laser scanning confocal microscope was used for fluorescence detection. The fluorescence values from randomly selected cells were analyzed with FV10-ASW Viewer Software.

### Western blot analysis

Western blot analysis was performed as previously described with slight modification [6]. Briefly, cells were cultured under experimental conditions for 24 h. Membrane proteins were extracted using the ProteoExtract Native Membrane Protein Extraction Kit (Calbiochem, San Diego, CA, USA) according to the manufacturer’s instructions. Anti-AQP1 rabbit polyclonal antibody H-55 clone (1:200; Santa Cruz Biotechnology Inc.) and horseradish peroxidase-conjugated antibody (Santa Cruz Biotechnology Inc.) were used as primary and secondary antibody, respectively. The intensity of the bands was quantified with ImageJ software (National Institutes of Health, Bethesda, MD, USA).

### RNA isolation and quantitative RT-PCR analysis

After incubation for 24 h under each experimental condition, total cellular RNA was isolated using the Sepasol extraction method (Sepasol-RNA I Super; Nacalai Tesque). All PCR analyses were performed with a LightCycler System (Roche Molecular Biochemicals, Indianapolis, IN, USA). Specific primers for PCR were as follows: endothelin-1 forward, 5′-AAGCCCTTCTAGGTCCAAGC-3′; endothelin-1 reverse, 5′-CGAGCTGCTGATGAAGACAC-3′; fibronectin forward, 5′-TATGACGATGGGAA-3′; fibronectin reverse, 5′-CTGTCAGCCTGTACA-3′; 18S forward, 5′-CTCAACACGGGAAACCTCAC-3′; and 18S reverse, 5′-AGACAAATCGCTCCACCAAC-3′.

### ELISA

After incubation for 24 h under experimental conditions, cell culture media were collected for enzyme-linked immunoabsorbent assay (ELISA). The secreted endothelin-1 levels in the culture supernatants were determined using an endothelin-1 ELISA kit (Enzo Life Sciences, Farmingdale, NY, USA) according to the manufacturer’s instructions.

### Detection of apoptosis by TUNEL

After incubation for 168 h under each condition, apoptotic nuclei were determined by direct immunoperoxidase detection of digoxigenin-labeled 3′DNA strand breaks using terminal deoxynucleotidyl transferase-mediated deoxyuridine triphosphate nick-end labeling (TUNEL) method. In situ end-labeling was performed using an Apoptosis In Situ Detection Kit (Wako) according to the manufacturer’s instructions.

### Measurement of intracellular ATP content

The intracellular ATP levels were measured by a commercially available intracellular ATP assay kit (Toyo Ink, Tokyo, Japan) according to the manufacturer’s instructions. The system can quantify intracellular ATP content by measuring the chemical luminescence emitted by the luciferase reaction in the presence of ATP and luciferine. After incubation for 24 h in glucose-free DMEM (Wako) with sodium pyruvate (Sigma), cells were incubated with or without 5 μM rotenone or 10μM antimycin A for 3 h under indicated experimental conditions. The amounts of chemical luminescence emitted for 400 ms due to the reaction were measured using a FilterMax F5 microplate reader with Multi-Mode Analysis software (Molecular Devices, Sunnyvale, CA, USA). The amount of ATP was determined from a standard curve based on given ATP solutions.

### Streptozotocin-induced diabetic mice

Vascular endothelial cell-specific MnSOD transgenic (eMnSOD-Tg) mice in C57BL/6 background were generated as described previously [9]. Male C57BL/6J mice were used as controls. Diabetes mellitus (DM) was induced in 8–10-week-old eMnSOD-Tg or control littermate male mice by an intraperitoneal injection of 120 mg/kg body weight streptozotocin (Wako) dissolved in citrate buffer, pH 4.5. Non-DM mice were injected with citrate buffer alone. After 4 weeks of buffer or streptozotocin injection, mice with blood glucose levels above 350 mg/dL and body weight loss less than 10% were used as the diabetic group. Animal care and use were in accordance with National Institutes of Health and institutional guidelines. The experimental procedures were approved by the ethics committee on animal experiments, Faculty of Life Sciences, Kumamoto University (Permit Number: A25-093).

### Immunohistochemistry

To detect tissue hypoxia, pimonidazole immunohistochemistry was performed as described previously [7]. Briefly, pimonidazole (60 mg/kg) was injected intraperitoneally 2 h before sacrifice. Mice were anesthetized with isoflurane inhalation, followed by an intraperitoneal injection of pentobarbital sodium (50 μg/g body weight). Anti-hypoxyprobe-1 FITC-mAb (1:100) and Alexa Fluor-488 Goat anti-mouse IgG (1:100) was used as primary and secondary antibody, respectively. A laser scanning confocal microscope was used for fluorescence detection. To detect AQP1 expression, tissue sections were prepared in the same manner as in the pimonidazole assay. Anti-AQP1 (1:100; Santa Cruz Biotechnology) was used as primary antibody and Alexa Fluor-594 Goat anti-rabbit IgG (1:100) was used as secondary antibody. Immunohistochemistry using anti-8-OHdG mouse monoclonal antibody (Japan Institute for the Control of Aging, Shizuoka, Japan) was performed according to the manufacturer’s instructions.

### Statistical analysis

Data are presented as mean ± SE. Statistical analysis was performed using one-way ANOVA followed by Tukey's multiple comparison test or Games–Howell multiple comparison test by SPSS software (version 21, IBM, USA). P values less than 0.05 were considered statistically significant differences.

## Results

### High glucose induced cellular hypoxia in endothelial cells and in glomeruli of diabetic mice

**Fig 1:**
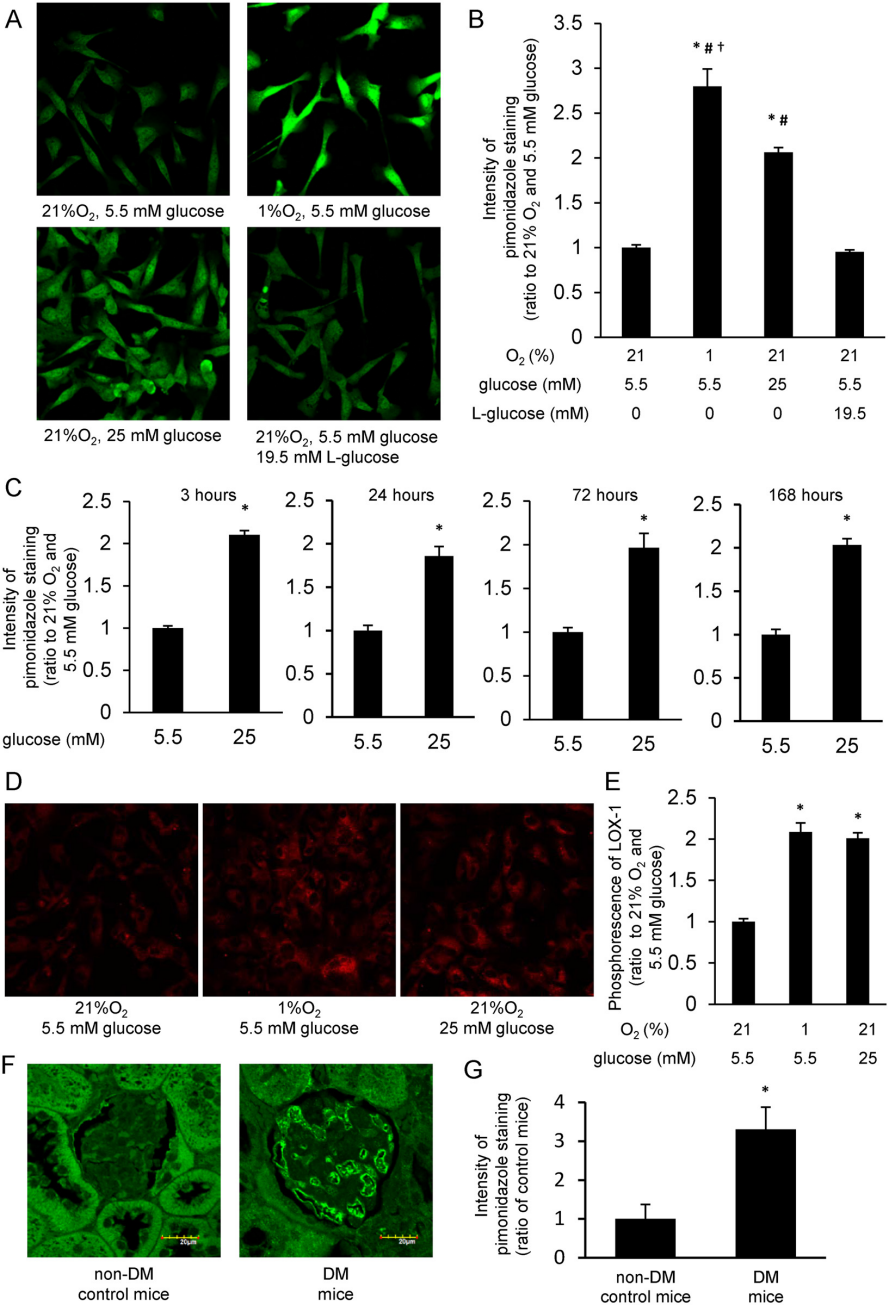
High-glucose induced cellular hypoxia was detected by pimonidazole and LOX-1 in BAECs and mice glomeruli. (A and B) Pimonidazole immunofluorescence of bovine aortic endothelial cells (BAECs). BAECs were incubated with the indicated conditions for 3 h in the presence of 10 μM pimonidazole (*green*). Relative intensities of pimonidazole staining were measured. *P < 0.05 compared with 21% O_2_ and 5.5 mM glucose; #P < 0.05 compared with 21% O_2_, 5.5 mM glucose and 19.5 mM L-glucose; †P < 0.05 compared with 21% O_2_ and 25 mM glucose. Data are five independent experiments in duplicate ± SEM. (C) Relative intensity of pimonidazole staining in cells incubated for various times at 21% O_2_. *P < 0.05 compared with 21% O_2_ and 5.5 mM glucose at each incubation time. Data from B and C are eight independent experiments in duplicate ± SEM. (D and E) Phosphorescence of hypoxia probe LOX-1. Cells were incubated with indicated conditions in the presence of 2 μM LOX-1 (*red*). Intensity of LOX-1 phosphorescence was measured. *P < 0.05 compared with 21% O_2_, 5.5 mM glucose. (F and G) Pimonidazole immunofluorescence of mouse glomeruli. Diabetes mellitus (DM) was induced by streptozotocin injection (120 mg/kg, intraperitoneally). Mice were studied at 4 weeks after DM induction. Intensity of pimonidazole staining (*green*) in glomeruli was measured. Scale bars represent 20 μm. *P < 0.05 compared with non-DM, control mice. n = 6/group.

To investigate oxygen tension within BAECs, we used pimonidazole, which is widely used to detect cellular and tissue hypoxia [10]. When cells were incubated with 1% O_2_ (hypoxic condition) and 5.5 mM glucose (normoglycemic condition) for 3 h, the intensity of pimonidazole staining was significantly increased compared with that in cells incubated with 21% O_2_ (normoxic condition) and 5.5 mM glucose (Fig 1A and 1B). Similarly, the intensity of pimonidazole staining in cells was significantly increased for 3 h incubation with 21% O_2_ and 25 mM glucose (hyperglycemic condition) compared with that in cells incubated with 21% O_2_ and 5.5 mM glucose. In contrast, the intensity of pimonidazole staining in cells was not altered by incubation with 21% O_2_ and 5.5 mM glucose plus 19.5 mM L-glucose, which was of equal osmotic pressure to 25 mM glucose. In addition, when cells were incubated with 21% O_2_ and 25 mM glucose for 3, 24, 72, or 168 h, increased intensity of pimonidazole staining persisted (Fig 1C). Meanwhile, the intensity of pimonidazole staining in cells incubated with 1% O_2_ and 25 mM glucose was comparable to that in cells incubated with 1% O_2_ and 5.5 mM glucose (S1 Fig).

To confirm hyperglycemia-induced cellular hypoxia, we used another hypoxia probe, LOX-1, which decreases in phosphorescence in response to the level of cellular oxygen tension [11]. Similarly to the results of pimonidazole staining, when cells were incubated for 24 h with either 1% O_2_ and 5.5 mM glucose or 21% O_2_ and 25 mM glucose, LOX-1 phosphorescence was significantly increased compared with that in cells incubated with 21% O_2_ and 5.5 mM glucose (Fig 1D and 1E).

To verify hyperglycemia-induced cellular hypoxia *in vivo*, we performed pimonidazole staining in glomeruli of control and streptozotocin (STZ)-induced DM mice. In STZ-induced DM mice, a significant increase in the intensity of pimonidazole staining in glomeruli was observed compared with that in non-DM control mice (Fig 1F and 1G).

### Cellular hypoxia is attenuated by inhibitors of mitochondrial electron transport chain or by MnSOD overexpression

**Fig 2:**
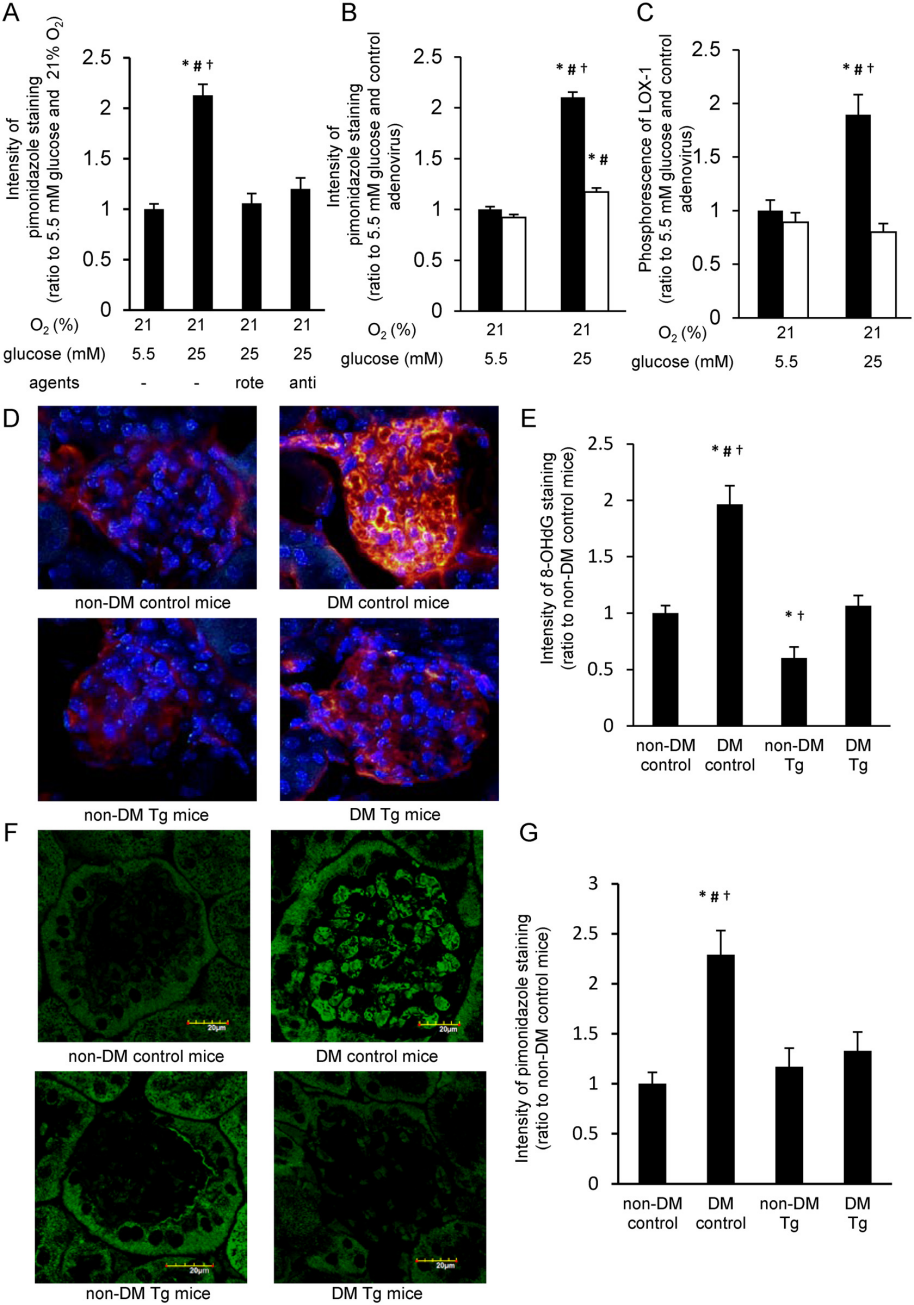
Cellular hypoxia was attenuated by inhibiting mitochondrial respiration and MnSOD overexpression *in vitro and vivo*. (A) Effect of mitochondrial respiratory inhibitors on cellular hypoxia. Cells were incubated for 3 h with indicated reagents (5 μM rotenone, 10 μM antimycin A) and 10 μM pimonidazole. Relative intensity of pimonidazole staining was measured. *P < 0.05 compared with 21% O_2_ and 5.5 mM glucose; #P < 0.05 compared with 21% O_2_, 5.5 mM glucose, and 5 μM rotenone; †P < 0.05 compared with 21% O_2_, 5.5 mM glucose and 10 μM antimycin A. Rote, rotenone; anti, antimycin A. Data are 10 independent experiments in duplicate ± SEM. (B) Effect of manganese superoxide dismutase (MnSOD) overexpression on pimonidazole staining. Cells transduced with MnSOD or control adenovirus were incubated for 3 h under indicated conditions and 10 μM pimonidazole, and relative intensity of pimonidazole staining was measured. Black bars = control adenovirus; white bars = MnSOD adenovirus. *P < 0.05 compared with 21% O_2_, 5.5 mM glucose, and control adenovirus; #P < 0.05 compared with 21% O_2_, 5.5 mM glucose, and MnSOD adenovirus; †P < 0.05 compared with 21% O_2_, 25 mM glucose, and MnSOD adenovirus. Data from B and C are eight independent experiments in duplicate ± SEM. (C) Effect of MnSOD overexpression on LOX-1 phosphorescence. Cells transduced with MnSOD or control adenovirus were incubated for 24 h under indicated conditions and 2 μM LOX-1, and the relative intensity of LOX-1 phosphorescence was measured. Black bars = control adenovirus; white bars = MnSOD adenovirus. *P < 0.05 compared with 21% O_2_, 5.5 mM glucose, and control adenovirus; #P < 0.05 compared with 21% O_2_, 5.5 mM glucose, and MnSOD adenovirus; †P < 0.05 compared with 21% O_2_, 25 mM glucose, and MnSOD adenovirus. (D-G) Immunofluorescence for 8-OHdG (8-hydroxy-2'-deoxyguanosine, D and E) and pimonidazole (F and G) in mice glomeruli (*blue*: 4′,6-diamidino-2-phenylindole; *red*: 8-OHdG; *green*: pimonidazole). Diabetes mellitus (DM) was induced in C57Bl/6 mice (8–10 weeks old) by streptozotocin injection. Immunohistochemistry was performed at 4 weeks after the onset of DM. Scale bars represent 20 μm. *P < 0.05 compared with non-DM control mice; #P < 0.05 compared with non-DM MnSOD-Tg mice; †P < 0.05 compared with diabetic MnSOD-Tg mice. n = 6/group

To assess whether hyperglycemia-induced cellular hypoxia is involved in oxygen consumption in mitochondria, we investigated the effect of inhibitors of mitochondrial electron transport chain, rotenone (an inhibitor of complex I) and antimycin A (an inhibitor of complex III). Increased intensity of pimonidazole staining by 3 h incubation with 25 mM glucose was completely blunted with either the treatment of rotenone or antimycin A (Fig 2A). There was no difference between 3 h incubation with 25 mM glucose and 5.5 mM glucose in intracellular ATP content. Meanwhile, both inhibitors decreased the intracellular ATP content by approximately 30 percent in high glucose condition (S2A and S2B Fig).

To evaluate the association of mtROS generation with hyperglycemia-induced cellular hypoxia, we investigated the effect of MnSOD overexpression on cellular hypoxia. In BAECs, both the increased intensity of pimonidazole staining and LOX-1 phosphorescence induced by 25 mM glucose were significantly suppressed by MnSOD overexpression (Fig 2B and 2C).

To confirm the effect of vascular specific MnSOD overexpression on the state of oxygen tension *in vivo*, we used eMnSOD-Tg mice, which specifically expressed MnSOD in endothelial cells by employing a Tie2 promoter/enhancer [9]. We evaluated oxidative stress in eMnSOD-Tg mice using 8-OHdG staining, which is a product of oxidative DNA damage that is used as a sensitive biomarker of intracellular oxidative stress [12]. The intensity of 8-OHdG staining in glomeruli of STZ-induced DM control mice was significantly increased compared with that of non-DM control mice. However, in STZ-induced DM eMnSOD-Tg mice, the 8-OHdG intensity in glomeruli was significantly suppressed (Fig 2D and 2E). Similarly to the results of 8-OHdG staining, increased intensity of pimonidazole staining was observed in glomeruli of DM control mice, while no increase was observed in DM eMnSOD-Tg mice (Fig 2F and 2G).

### Involvement of AQP1 in cellular oxygen tension

To investigate the mechanism by which MnSOD overexpression could attenuate cellular hypoxia, we examined the membrane expression of AQP1, which was reported to function as a water and gas channel in mouse endothelial cells [13].

**Fig 3:**
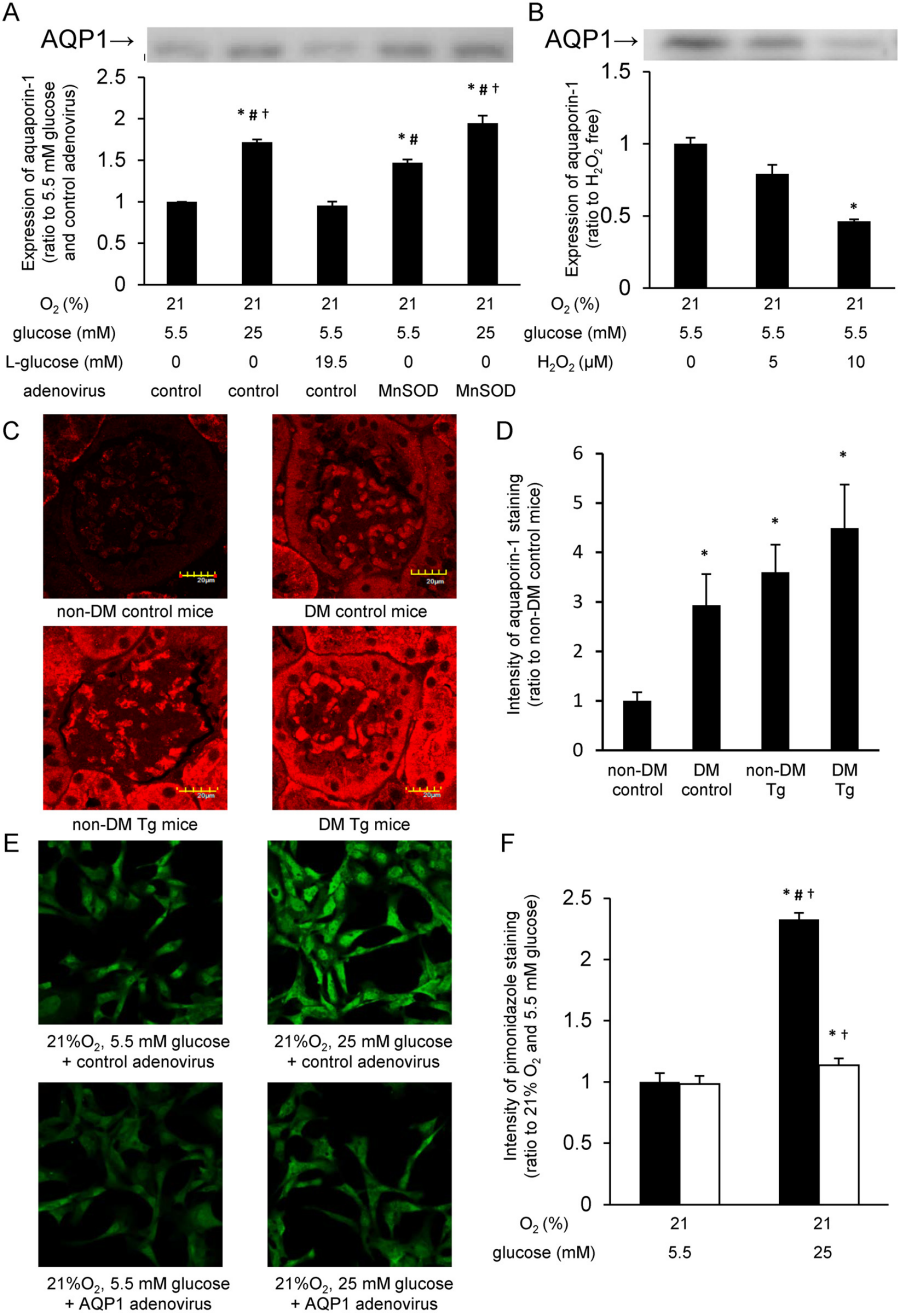
AQP1 is involved in high glucose-induced cellular hypoxia and is affected by mitochondrial ROS generation. (A) Expression levels of membrane aquaporin-1 (AQP1) protein. Cells transduced with manganese superoxide dismutase (MnSOD) or control adenovirus were incubated under indicated conditions for 24 h, and membrane proteins were examined by western blotting for detection of membrane AQP1 expression. The experiments were repeated at least four times. *P < 0.05 compared with 5.5 mM glucose and control adenovirus; #P < 0.05 compared with 5.5 mM glucose, 19.5 mM L-glucose, and control adenovirus; †P < 0.05 compared with 5.5 mM glucose, MnSOD adenovirus. Data from A and B are four independent experiments in duplicate ± SEM. (B) Hydrogen peroxide attenuated membrane AQP1 expression. Cells were incubated under indicated conditions for 30 min, and membrane proteins were subjected to western blotting. The experiments were repeated at least four times. *P < 0.05 compared with no agent. (C and D) Immunofluorescence for AQP1 in mouse glomeruli (*red*: AQP1). C57Bl/6 mice (8 weeks old) were made diabetes mellitus (DM) by streptozotocin injection. Immunohistochemistry was performed at 4 weeks after the onset of DM. Scale bars represent 20 μm. *P < 0.05 compared with non-DM control mice. n = 5/group. (E and F) Effect of AQP1 overexpression on pimonidazole staining. Cells infected with AQP1 or control adenovirus were incubated for 3 h, and relative intensity of pimonidazole staining (*green*) was measured. Black bars = control adenovirus; white bars = AQP1 adenovirus. *P < 0.05 compared with 21% O_2_, 5.5 mM glucose, and control adenovirus; #P < 0.05 compared with 21% O_2_, 25 mM glucose, and AQP1 adenovirus; †P < 0.05 compared with 21% O_2_, 5.5 mM glucose, and AQP1 adenovirus. Data are eight independent experiments in duplicate ± SEM.

Western blot analysis showed that incubation with 25 mM glucose for 24 h increased the expression of AQP1 protein in the membrane fraction compared with that with 5.5 mM glucose, whereas incubation with 5.5 mM glucose plus 19.5 mM L-glucose did not affect the expression of AQP1 protein. The expression of AQP1 protein was also increased by MnSOD overexpression compared with cells infected with control adenovirus and incubated with 5.5 mM glucose (Fig 3A). A significant additive effect of MnSOD overexpression and 25 mM glucose on AQP1 expression was not observed.

As mtROS was decreased by MnSOD overexpression, the effect of ROS on the expression of membrane AQP1 was examined. When cells were incubated with 10 μM H_2_O_2_ for 30 min, the expression level of membrane AQP1 was significantly decreased compared with controls (Fig 3B).

To verify the effect of MnSOD on AQP1 expression *in vivo*, we examined the expression levels of AQP1 in glomeruli of control and eMnSOD-Tg mice. The expression of AQP1 in glomeruli was significantly increased in DM control mice compared with that in non-DM control mice. The expression of AQP1 in glomeruli in non-DM eMnSOD-Tg mice was also increased compared with that in non-DM control mice. The expression of AQP1 in glomeruli of DM eMnSOD-Tg mice showed a trend towards being higher than that of non-DM eMnSOD-Tg mice, but it was not statistically significant (Fig 3C and 3D).

To ensure that AQP1 actually facilitated O_2_ diffusion across the membrane in endothelial cells, we used BAECs infected with adenovirus expressing AQP1. The increased intensity of pimonidazole staining induced by 25 mM glucose for 3 h was significantly suppressed by AQP1 overexpression (Fig 3E and 3F).

### AQP1 overexpression attenuated the hyperglycemia-induced phenomena

**Fig 4:**
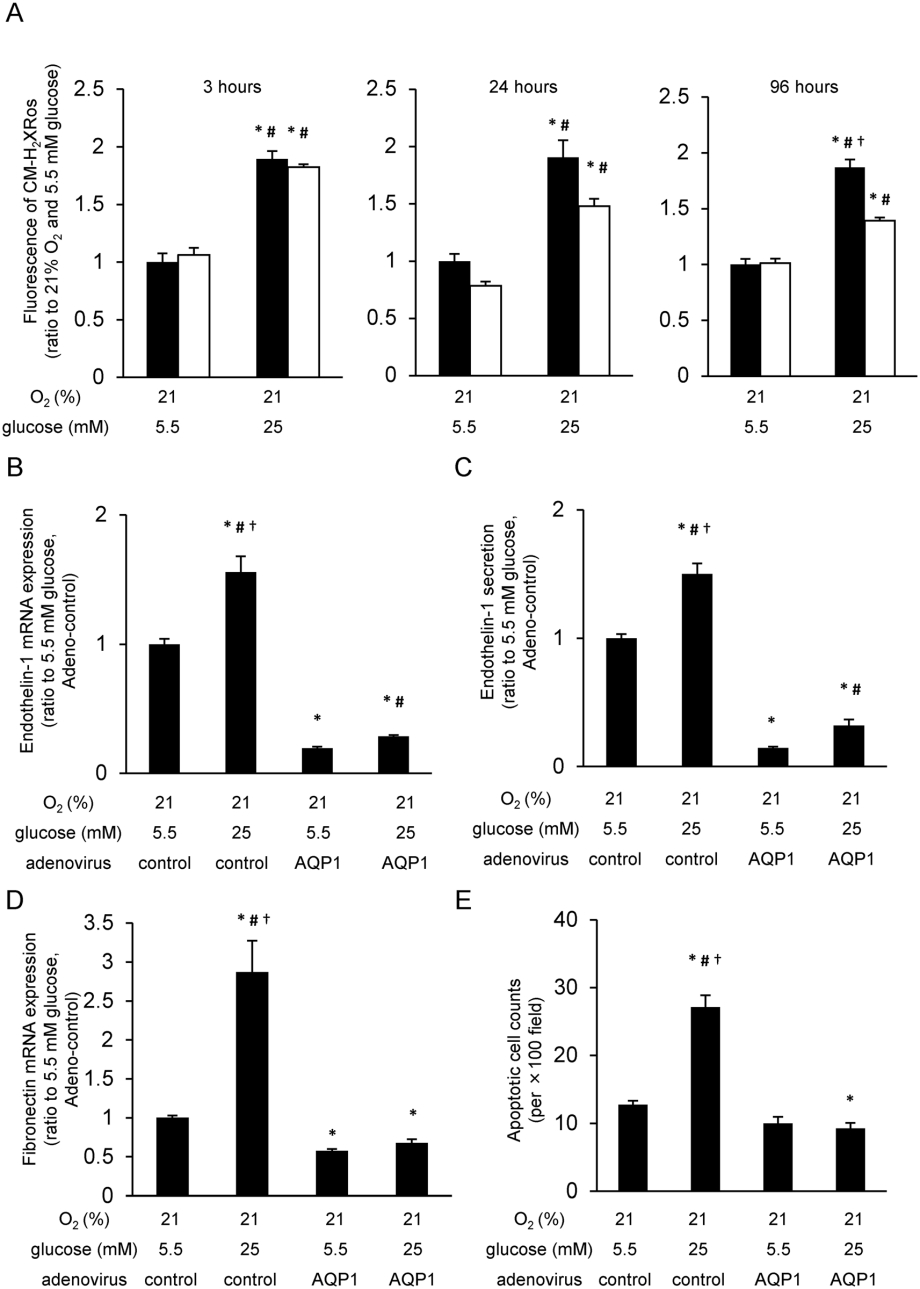
AQP1 overexpression decreased high glucose-induced mitochondrial ROS generation and high glucose-induced phenomena. (A) Mitochondrial reactive oxygen species (mtROS) generation in aquaporin-1 (AQP1) overexpression cells. Cells were incubated under indicated conditions, and treated with 300 nM CM-H_2_XRos for 15 min. Relative intensity of fluorescence of CM-H_2_XRos was measured. Black bars = control adenovirus; white bars = AQP1 adenovirus. *P < 0.05 compared with 5.5 mM glucose and control adenovirus; #P < 0.05 compared with 5.5 mM glucose and AQP1 adenovirus; †P < 0.05 compared with 25 mM glucose and AQP1 adenovirus. Data are eight independent experiments in duplicate ± SEM. (B) Effect of AQP1 overexpression on endothelin-1 mRNA expression. Cells were incubated under indicated conditions for 24 h. The expression levels of endothelin-1 mRNA were measured by quantitative RT-PCR analysis. *P < 0.05 compared with 21% O_2_, 5.5 mM glucose, and control adenovirus; #P < 0.05 compared with 21% O_2_, 5.5 mM glucose, and AQP1 adenovirus; †P < 0.05 compared with 21% O_2_, 25 mM glucose, and AQP1 adenovirus. Data are five independent experiments in duplicate ± SEM. (C) Effect of AQP1 overexpression on endothelin-1 secretion. Cells were incubated under indicated conditions for 24 h, and endothelin-1 secretion was measured by ELISA assay. *P < 0.05 compared with 21% O_2_, 5.5 mM glucose, and control adenovirus; #P < 0.05 compared with 21% O_2_, 5.5 mM glucose, and AQP1 adenovirus; †P < 0.05 compared with 21% O_2_, 25 mM glucose, and AQP1 adenovirus. Data are six independent experiments in duplicate ± SEM. (D) Effect of AQP1 overexpression on fibronectin mRNA expression. Cells were incubated under indicated conditions for 24 h. The expression levels of fibronectin mRNA were measured by quantitative RT-PCR analysis. *P < 0.05 compared with 21% O_2_, 5.5 mM glucose, and control adenovirus; #P < 0.05 compared with 21% O_2_, 5.5 mM glucose, and AQP1 adenovirus; †P < 0.05 compared with 21% O_2_, 25 mM glucose, and AQP1 adenovirus. Data are five independent experiments in duplicate ± SEM. (E) Effect of AQP1 overexpression on cell apoptosis. Cells were incubated under indicated conditions for 168 hours. Data are expressed as the mean number of positive cells/section of 10 independent sections ± SEM. *P < 0.05 compared with 21% O_2_, 5.5 mM glucose, and control adenovirus; #P < 0.05 compared with 21% O_2_, 5.5 mM glucose, and AQP1 adenovirus; †P < 0.05 compared with 21% O_2_, 25 mM glucose, and AQP1 adenovirus.

We examined the effect of AQP1 overexpression on hyperglycemia-induced mtROS generation. The increased fluorescence of CM-H_2_XRos observed by incubation with high glucose (25 mM glucose) for 3 or 24 h was not affected by AQP1 overexpression. Contrary, after 96 h of incubation, the increased fluorescence of CM-H_2_XRos induced by 25 mM glucose was significantly suppressed by AQP1 overexpression (Fig 4A). To clear the involvement of AQP1 on hyperglycemia-induced ROS generation, we also investigated the effect of AQP1 overexpression on 8-OHdG formation in BAECs. There was no significant difference on the intensity of 8-OHdG staining between 24 h incubation with 5.5 mM glucose and that with 25 mM glucose. On the other hand, the intensity of 8-OHdG staining by 96 h incubation with 25 mM glucose was significantly higher than that with 5.5 mM glucose. Moreover, the increased 8-OHdG staining by 25 mM glucose was significantly suppressed by AQP1 or MnSOD overexpression (S3A and S3B Fig).

As endothelin-1 and fibronectin overproduction and apoptosis are characteristic features in both hyperglycemia [3] and hypoxia [4], we evaluated the effect of AQP1 overexpression on these phenomena. Endothelin-1 mRNA induction, endothelin-1 secretion, and fibronectin mRNA induction were increased by incubation with 25 mM glucose for 24 h compared with those with 5.5 mM glucose. These high glucose-induced phenomena were significantly suppressed by AQP1 overexpression (Fig 4B–4D). Similarly, when cells were incubated with 25 mM glucose for 168 h, apoptosis was increased compared with that of 5.5 mM glucose. However, the increased apoptosis by high glucose was also significantly suppressed by AQP1 overexpression (Fig 4E).

## Discussion

We hypothesized that hyperglycemia causes cellular hypoxia, and that hyperglycemia-induced cellular hypoxia and mtROS generation promotes hyperglycemic damage in a coordinated manner.

We first confirmed that pimonidazole staining was increased in endothelial cells by the incubation of high glucose, and it was also increased in glomeruli of diabetic mice. Consistent with our findings, pimonidazole staining was increased in pancreatic β cells by hyperglycemia [7]. However, it is important to note that the pyridine nucleotide redox state might affect pimonidazole adduct formation, because both NADH and NADPH can act as electron donors for the formation of the pimonidazole nitro radical anion [14]. An earlier study proposed that increased cytosolic ratio of free NADH/NAD^+^ caused by hyperglycemia is a characteristic feature of poorly controlled diabetes, and that this increased NADH/NAD^+^ ratio plays an important role in the pathogenesis of diabetic complications [15]. This phenomenon was called “pseudohypoxia” because impaired oxidation of NADH to NAD^+^ and the resulting increased NADH/NAD^+^ ratio was observed in hypoxic tissues. Therefore, we are concerned that an increase of pimonidazole staining by hyperglycemia may reflect “pseudohypoxia.” However, it was reported that the pyridine nucleotide redox state did not determine the rate of reductive metabolism of pimonidazole, and instead, the cellular oxygen tension regulated this process [14]. Additionally, we reconfirmed that hyperglycemia induced cellular hypoxia using a novel hypoxia-sensing probe, LOX-1 [11]. LOX-1 is a phosphorescent light-emitting iridium complex. Phosphorescence of LOX-1 is quenched by molecular oxygen and is increased in response to low levels of oxygen. It is inconceivable that the pyridine nucleotide redox state affects phosphorescence of LOX-1. Therefore, increases of pimonidazole staining and LOX-1 phosphorescence observed in the present study suggested that hyperglycemia induced “true cellular hypoxia” in endothelial cells. To the best of our knowledge, our study is the first to investigate the involvement of high glucose-induced “true cellular hypoxia” in the pathogenesis of diabetes complications.

We next examined which mechanisms are involved in hyperglycemia-induced cellular hypoxia. We confirmed that rotenone and antimycin A, both of which are inhibitors of the mitochondrial electron transport chain, suppressed hyperglycemia-induced cellular hypoxia since it was reported that these inhibitors can suppress oxygen consumption in mitochondria in pancreatic β cells [7],

We previously proposed that hyperglycemia-induced mtROS generation was a key event in the development of diabetic complications [1, 9, 16]. Therefore, we examined the association between mtROS generation and high glucose-induced cellular hypoxia. In this study, we found the following: (a) overexpression of MnSOD suppressed hyperglycemia-induced cellular hypoxia in BAECs and in glomeruli of mice; (b) the expression level of AQP1 was increased by overexpression of MnSOD in BAECs and in glomeruli of mice; (c) hydrogen peroxide decreased the expression level of AQP1 in BAECs; (d) overexpression of AQP1 suppressed hyperglycemia-induced cellular hypoxia in BAECs; and (e) hyperglycemia-induced endothelin-1 mRNA induction, endothelin-1 secretion, fibronectin mRNA induction, and apoptosis—all of which are characteristic features in both hyperglycemia [3] and hypoxia [4]—were suppressed by the overexpression of AQP1 in BAECs. Consistent with these findings, it was reported that oxygen permeability of lipid bilayers was much lower than previously thought [17], and AQP1, which was known as a water channel, also facilitated oxygen diffusion across the cell membrane [13]. Additionally, the expression level of AQP1 in the pulmonary capillary endothelium of mice was decreased by lipopolysaccharide, an inducer of ROS [18]. Furthermore, α-lipoic acid, an antioxidant, attenuated the downregulation of AQP1 in response to ischemia-reperfusion in rat kidneys [19], and tempol, another antioxidant, normalized renal hypoxia observed in mice with 5/6 surgical reduction of renal mass [20].

Taken together, it appears that close associations exist among cellular hypoxia, AQP1 expression, and mtROS generation. The question thus arises of how MnSOD overexpression and high glucose induce AQP1 expression in endothelial cells. Furthermore, it is unclear whether increased expression of AQP1 by MnSOD overexpression is sufficient to suppress cellular hypoxia, because high glucose also increased the expression of AQP1 to the similar degree as MnSOD overexpression. Prior to our study, Madonna et al. have reported that the increased AQP1 expression by high glucose may lead to the progression of vascular injury in diabetes, not to the prevention [21, 22]. The mechanisms underlying this difference between our study and Madonna’s are unclear at this moment.

To answer these questions, further studies of transcriptional and posttranscriptional regulation of the AQP1 gene, functional analysis of AQP1, and expression analysis of other types of AQP will be required. Future *in vivo* investigations using AQP1 overexpression or knockdown mice may be useful to determine the therapeutic utility of AQP1 in diabetes. However, it is important to note that AQP1 may serve as a molecular target to prevent diabetic complications because hyperglycemia-induced endothelin-1 and fibronectin overproduction and apoptosis were all suppressed by overexpression of AQP1.

Interestingly, increased mtROS generation for 3 or 24 h incubation with high glucose was not inhibited by the overexpression of AQP1, although that of 96 h incubation was significantly inhibited. The reasons underlying the different effects of AQP1 overexpression on mtROS generation by the incubation time are unknown. However, these findings suggest distinct mechanisms of mtROS generation by hyperglycemia exist depending on the duration of hyperglycemia. Further study will be required.

**Fig 5:**
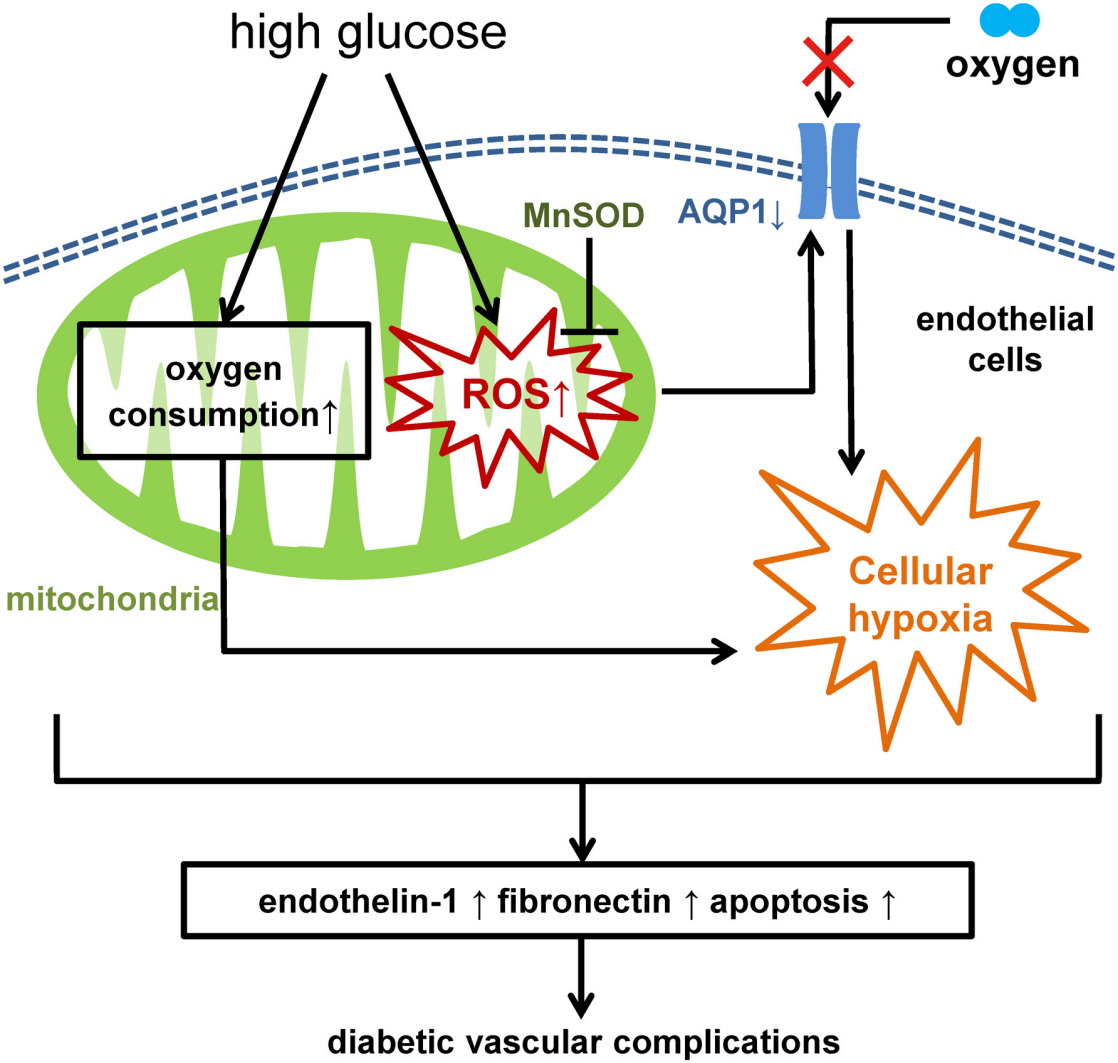
Proposed model of the pathogenesis of diabetic complications. High glucose increases mitochondrial reactive oxygen species (mtROS) generation. High glucose also induces cellular hypoxia through increased O_2_ consumption in mitochondria. Cellular hypoxia may also be affected through suppressed aquaporin-1 (AQP1) expression induced by mtROS generation. Hyperglycemia-induced cellular hypoxia and mtROS generation may simultaneously promote hyperglycemic damage including overproduction of endothelin-1 and fibronectin, and induction of apoptosis, which leading to diabetic vascular complications.

The results from this study demonstrated the following: (a) high glucose caused true cellular hypoxia; (b) high glucose may increase oxygen consumption in mitochondria; (c) cellular hypoxia may also be affected by mtROS generation and AQP1 expression; (d) overexpression of AQP1 suppressed high glucose-induced cellular hypoxia and other high glucose-induced phenomena. Therefore, it was suggested that hyperglycemia-induced cellular hypoxia and mtROS generation may promote hyperglycemic damage in a coordinated manner (Fig 5). Our findings also suggest that AQP1 could be a potential molecular target for the novel pharmacological approaches to prevent diabetic vascular complications.

## Supporting Information

S1 Fig Hyperglycemia did not enhanced the intensity of pimonidazole at 1% oxygen tension in BAECs.fig. Pimonidazole immunofluorescence of bovine aortic endothelial cells (BAECs). BAECs were incubated with the indicated conditions for 3 h at 1 or 21% O_2_ in the presence of 10 μM pimonidazole. Relative intensity of pimonidazole staining were measured. *P < 0.05 compared with 21% O_2_ and 5.5 mM glucose. Data are eight independent experiments in duplicate ± SEM.
(TIFF)

S2 Fig Mitochondrial respiratory blockades decreased the intracellular ATP content in high glucose condition. (A) Effect of high glucose on the intracellular ATP content of bovine aortic endothelial cells (BAECs). Cells were incubated for 3 h with 5.5 or 25 mM glucose. The intracellular ATP levels were assessed by measuring amounts of the chemical luminescence emitted by the luciferine/luciferase reaction. Data are seven independent experiments in duplicate ± SEM. (B) Effect of mitochondrial respiratory blockades on the intracellular ATP content of BAECs in high glucose condition. Cells were treated for 3 h with indicated reagents (5 μM rotenone, 10 μM antimycin A). *P < 0.05 compared with 21% O_2_ and 25 mM glucose, no reagent. rote, rotenone; anti, antimycin A. Data are four independent experiments in duplicate ± SEM.
(TIFF)

S3 Fig AQP1 overexpression decreased the high glucose-induced 8-OHdG formation *in vitro*. (A) 8-OHdG (8-hydroxy-2'-deoxyguanosine) immunofluorescence of bovine aortic endothelial cells (BAECs). Cells were incubated with 5.5 or 25 mM glucose for 24 h. Relative intensities of 8-OHdG staining were measured. Data are eight independent experiments in duplicate ± SEM. (B) Effect of AQP1 overexpression on high-glucose induced 8-OHdG formation. Cells were incubated under indicated conditions for 96 h. Relative intensities of 8-OHdG staining were measured. *P < 0.05 compared with 21% O_2_, 25 mM glucose, and control adenovirus. Data are eight independent experiments in duplicate ± SEM.
(TIFF)
