# Supplementary figures and images for: Hyperglycemia Induces Cellular Hypoxia through Production of Mitochondrial ROS Followed by Suppression of Aquaporin-1
Source: PLoS One. 2016 Jul 6;11(7):e0158619. doi: 10.1371/journal.pone.0158619 (PMC4934928; doi:10.1371/journal.pone.0158619)

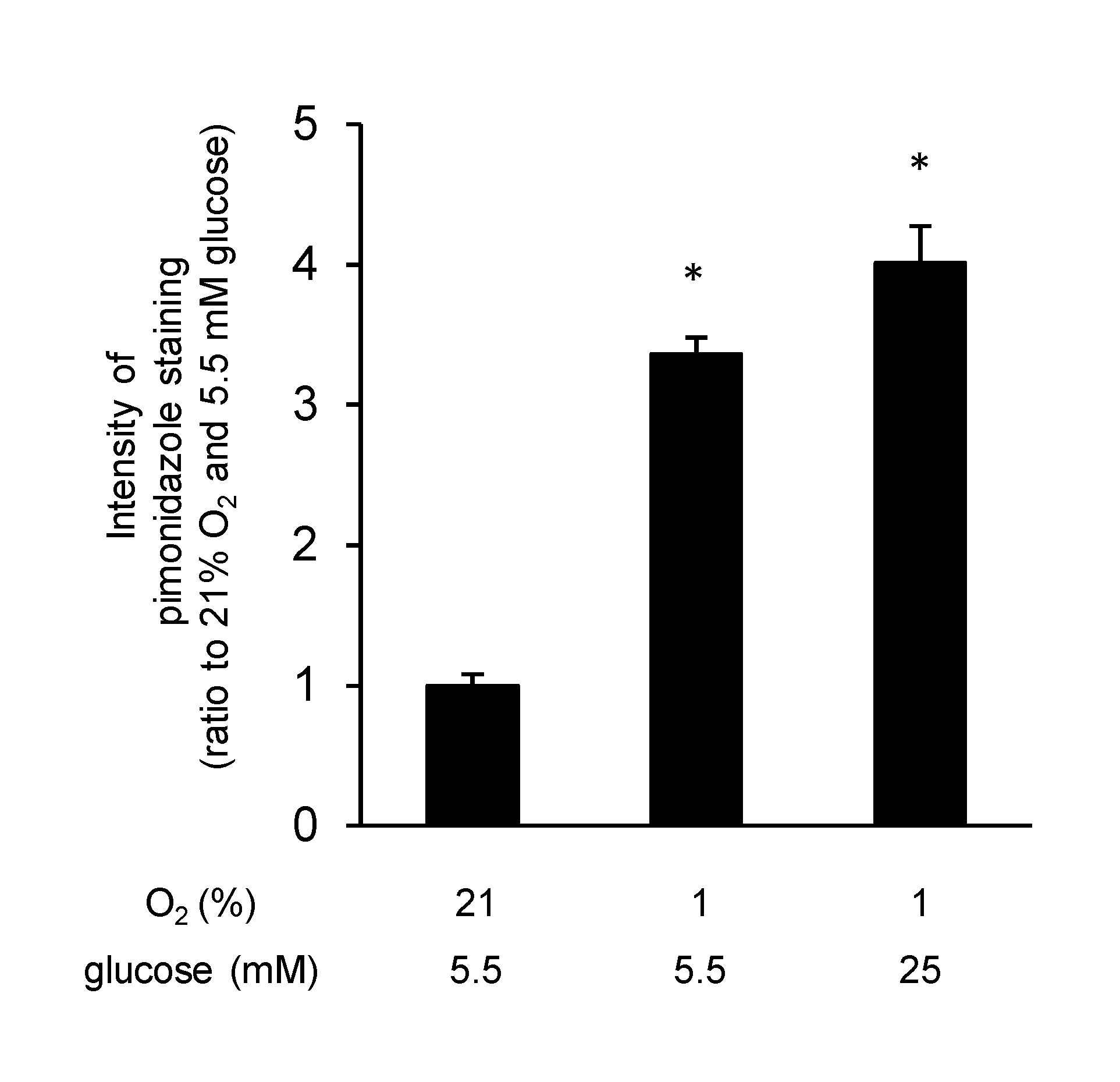

Supplement: S1 Fig — Pimonidazole immunofluorescence of bovine aortic endothelial cells (BAECs). BAECs were incubated with the indicated conditions for 3 h at 1 or 21% O2 in the presence of 10 μM pimonidazole. Relative intensity of pimonidazole staining were measured. *P < 0.05 compared with 21% O2 and 5.5 mM glucose. Data are eight independent experiments in duplicate ± SEM. (TIFF) [file pone.0158619.s001.tiff]

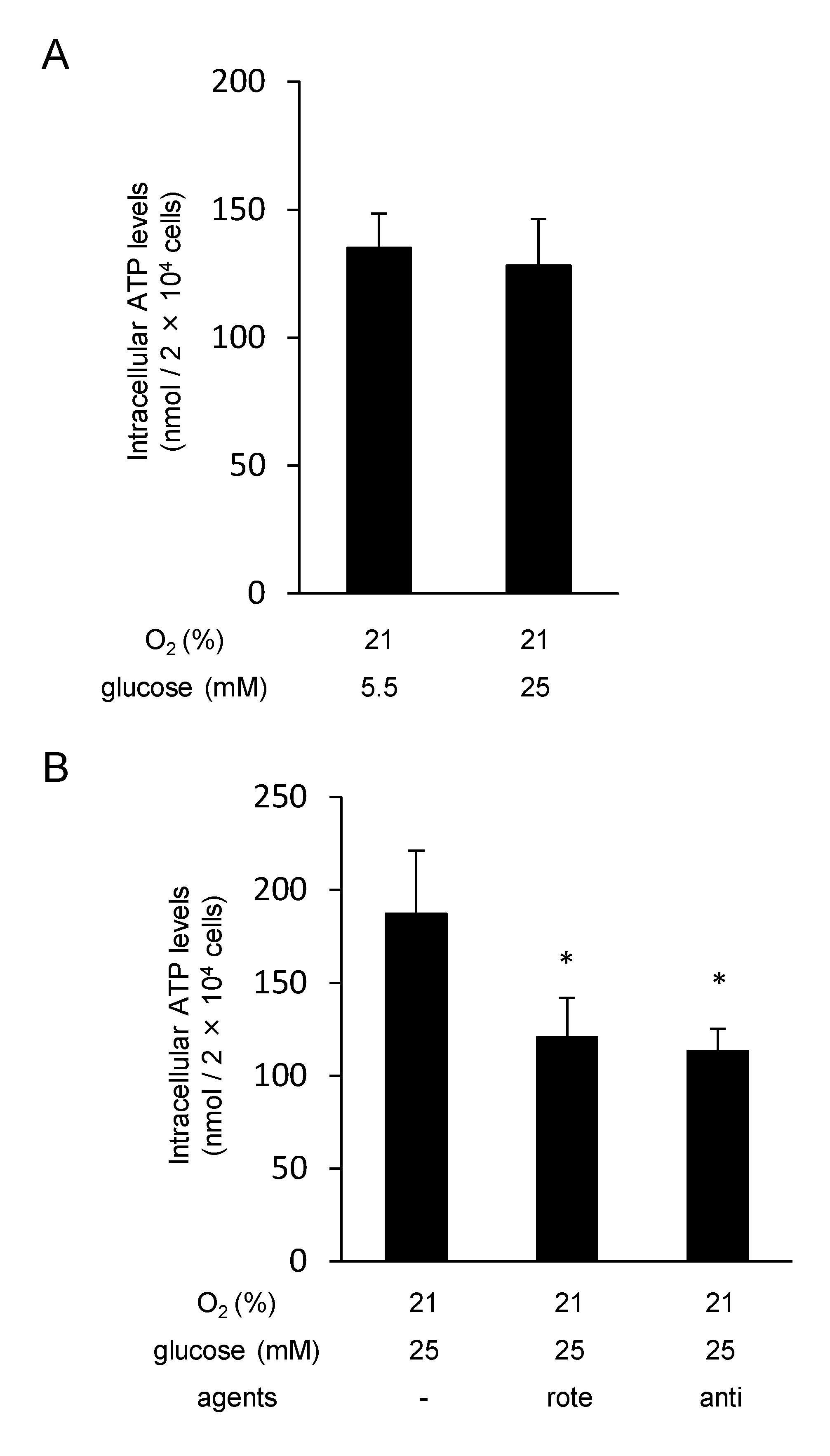

Supplement: S2 Fig — (A) Effect of high glucose on the intracellular ATP content of bovine aortic endothelial cells (BAECs). Cells were incubated for 3 h with 5.5 or 25 mM glucose. The intracellular ATP levels were assessed by measuring amounts of the chemical luminescence emitted by the luciferine/luciferase reaction. Data are seven independent experiments in duplicate ± SEM. (B) Effect of mitochondrial respiratory blockades on the intracellular ATP content of BAECs in high glucose condition. Cells were treated for 3 h with indicated reagents (5 μM rotenone, 10 μM antimycin A). *P < 0.05 compared with 21% O2 and 25 mM glucose, no reagent. rote, rotenone; anti, antimycin A. Data are four independent experiments in duplicate ± SEM. (TIFF) [file pone.0158619.s002.tiff]

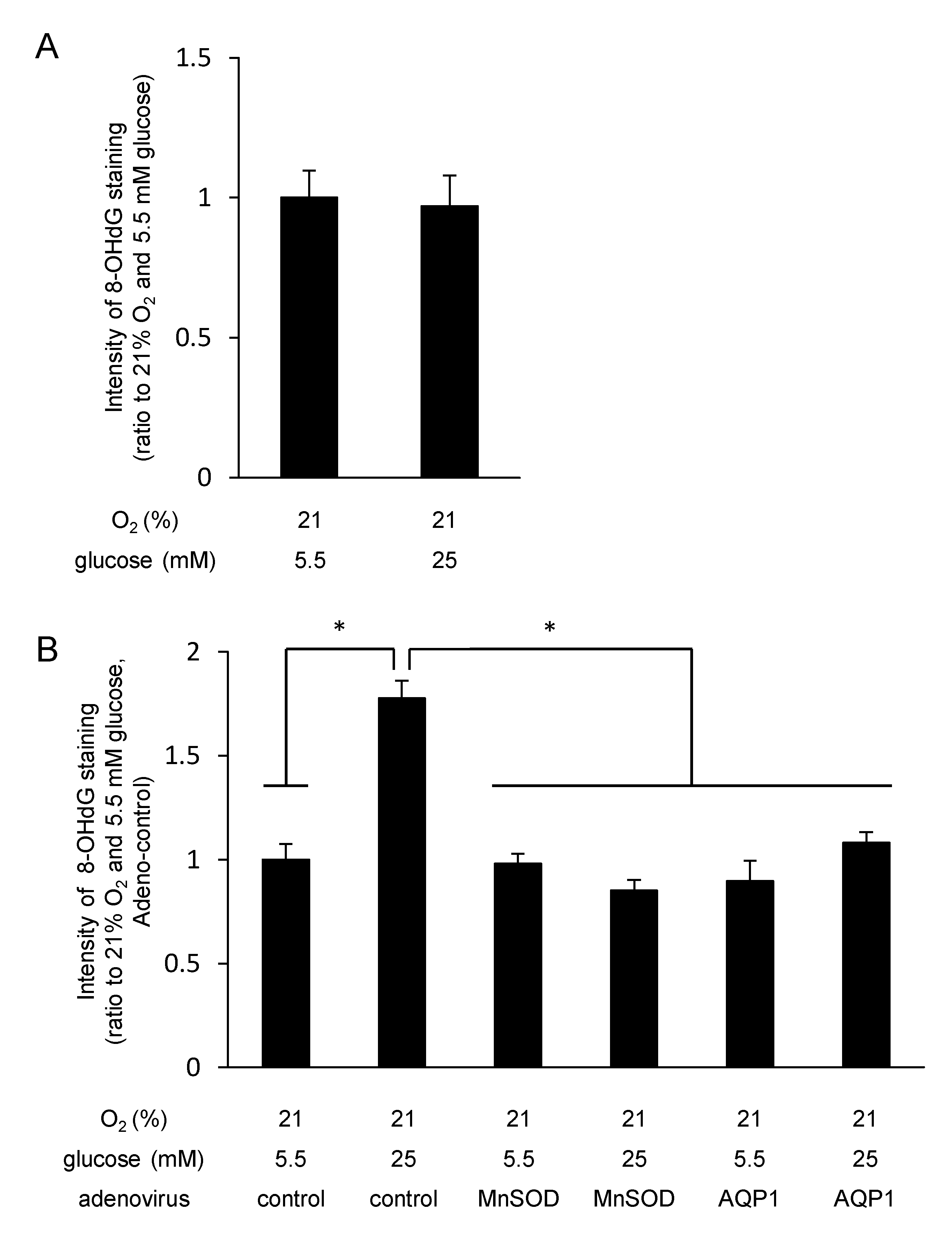

Supplement: S3 Fig — (A) 8-OHdG (8-hydroxy-2'-deoxyguanosine) immunofluorescence of bovine aortic endothelial cells (BAECs). Cells were incubated with 5.5 or 25 mM glucose for 24 h. Relative intensities of 8-OHdG staining were measured. Data are eight independent experiments in duplicate ± SEM. (B) Effect of AQP1 overexpression on high-glucose induced 8-OHdG formation. Cells were incubated under indicated conditions for 96 h. Relative intensities of 8-OHdG staining were measured. *P < 0.05 compared with 21% O2, 25 mM glucose, and control adenovirus. Data are eight independent experiments in duplicate ± SEM. (TIFF) [file pone.0158619.s003.tiff]
